# The Demographic Data and Prevalence of Thromboembolic Events Among Inflammatory Bowel Disease Patients in Buraydah, Al-Qassim Region

**DOI:** 10.7759/cureus.29108

**Published:** 2022-09-13

**Authors:** Arwa A Alodheilah, Omar A Alnujeidi, Nada A AlDhuwayhi, Maha M AlDhilan, Fatimah S Alsultan, Majd I Aldhuwayhi, Haya S Alnumayr, Fai M AlHotan, Shatha E Aljamaan

**Affiliations:** 1 Medicine, Qassim University, Qassim, SAU; 2 Hematology, King Fahad Specialist Hospital, Qassim, SAU; 3 Internal Medicine, King Fahad Specialist Hospital, Qassim, SAU

**Keywords:** al-qassim, inflammatory bowel disease, thromboembolic events, prevalence, demographic data

## Abstract

Introduction

Inflammatory bowel diseases (IBD) (Crohn’s disease (CD) and ulcerative colitis (UC)) are considered among the commonest gastrointestinal (GI) tract diseases manifesting with chronic, recurring episodes of gut inflammation, especially in the colon. Each disease has its pattern, symptoms, severity of pain, extension, management, and prognosis. However, these diseases share most of the various complications, including the GI tract and extending it to other systems such as musculoskeletal, skin, liver, and pulmonary systems.

Objectives

We aim to identify the demographic data, prevalence, risk factors, clinical presentation, and management (medications given and investigations ordered) of thromboembolic events (TEE) among inflammatory bowel disease patients at King Fahad Specialist Hospital (KFSH) in Buraydah, Al-Qassim, Saudi Arabia.

Materials and methods

This is a retrospective, cross-sectional study. All included patients with IBD who meet the inclusion criteria between January 2020 and January 2022 in KFSH were reviewed, and data were analyzed using the Statistical Package for the Social Sciences (SPSS) Statistics version 23.0 (IBM Corp., Armonk, NY, USA).

Results

A total of 187 participants were included in the current study. The mean age of the participants ± standard deviation (SD) was 28.7 ± 10.8 years old. Of the participants, 107 (57.2%) were males. A total of 121 (64.7%) participants were diagnosed with Crohn’s disease (CD), 56 (29.9%) with ulcerative colitis (UC), and 10 (5.3%) with both CD and UC. In 156 (83%) participants, the duration of the disease was 1-5 years. Among the IBD patients, two (1.1%) had TEE in the interval resolution middle and left portal vein, as well as the inferior mesenteric vein. The majority of the participants (73.3%) were with no history of comorbid conditions. The most reported clinical symptoms were chest pain as reported by 3.2% of the participants. Abdominal computed tomography (CT) was the most reported method of diagnosis as reported by 35.8% of the participants. Of the participants, 8.6% used heparin prophylactically, 0.5% used heparin as a treatment, and 0.5% used enoxaparin as a treatment. Moreover, 20.3% of the participants used prophylactic treatment, whereas about 79.7% did not use prophylactic treatment. Old age, extensive disease, colorectal surgery, and pregnancy were not found to be associated with thromboembolic events (p = 1.000, 0.400, 0.164, and 0.053, respectively). Age, gender, and nationality were not significantly associated with thromboembolic events (p = 0.915, 1.000, and 1.000, respectively).

Conclusion

Despite IBD being one of the emerging health concerns in the Kingdom of Saudi Arabia, records showed that the prevalence of thromboembolic events was found to be lower when compared to the prevalence reported in the relevant multinational studies. The was no difference in factors affecting the development of thromboembolic events between IBD patients and the general population.

Recommendations

We should stress raising awareness of IBD patients about their condition, the increased risks of developing thromboembolic events, and the proper prevention methods.

## Introduction

Inflammatory bowel disease (IBD) refers to two disorders, Crohn’s disease (CD) and ulcerative colitis (UC), which represent chronic inflammation of the gastrointestinal (GI) tract. The inflammatory process is associated with a hypercoagulable state and an increased risk for venous thromboembolism (VTE), and thromboembolic disease (TED) is still the primary cause of death in IBD patients [1]. Many risk factors can influence the progression of thrombotic events in IBD patients, such as pregnancy, medications, disease activity, and genetics [2]. Therefore, the influence of TED on IBD prognosis and therapeutic management necessitates the development of strategies to identify IBD patients who are very susceptible to TED.

Furthermore, using health administrative data from Ontario, Canada, a population-based cohort study was conducted between 2002 and 2016 to determine the incidence and timing of VTE in the post-discharge setting in patients with IBD and if IBD increases the risk of post-discharge VTE in medical and surgical patients. There were 81,900 IBD discharges (62,848 nonsurgical and 19,052 surgeries) matched to non-IBD controls. Patients with nonsurgical IBD had a cumulative incidence of VTE of 2.3%, and surgical IBD patients had a cumulative incidence of 1.6% at 12 months following discharge. Nonsurgical IBD patients and surgical patients with ulcerative colitis are 1.7-fold more likely than non-IBD patients to have VTE after discharge. These findings highlight the importance of increased vigilance and consideration of thromboprophylaxis in this population [3].

In addition, a prospective multicenter cohort study was done among 85 patients hospitalized in three gastrointestinal centers between 2013 and 2018 [4]. The incidence of VTE in individuals with IBD (n = 42) was compared to patients with other digestive illnesses (n = 43). VTE was 16.7% more common in people with IBD than in other digestive illnesses [4]. VTE was seen in six of 22 patients with ulcerative colitis (27.2%) and only one of 20 individuals with Crohn’s disease among IBD patients (5%). VTE was discovered in four IBD patients at the time of admission and three patients two weeks later [4].

A retrospective study was done among 100 patients with IBD in Tunisia between January 2012 and December 2018 to measure the prevalence of thromboembolic event (TEE) in IBD patients and determine epidemiological, clinical, and evolutionary characteristics; TEE was found in 5.9% of patients with IBD [5]. These patients, divided into four women and two men, had an average age of 41 years old. One patient had ulcerative colitis, and five others had Crohn’s disease. All of the patients had active IBD [5].

In another study in Asian countries, a nationwide survey was conducted in Japan to determine mortality and the risk of progressing severity of IBD disease due to arterial and venous thromboembolism (TE). Patients with and without severe TE and TE-related death were compared on their features, laboratory results, state of therapy, and environment at the time TE developed. TE was detected in 1.89% of the 31,940 IBD patients. Severe TE and TE-related mortality were detected in 10.7% of all IBD patients and 1% of TE with IBD patients, respectively. In Asia, therapeutic and preventative therapies for IBD-associated TE are urgently needed [6].

In the local aspect, a study was done among 100 Saudi patients in Riyadh in 2016 to examine the risk factors for TEE in IBD in a population with prevalent consanguinity [1]. There were 51 (51%) women in the group, with a mean standard deviation (SD) of 31.24 ± 10.78 years. Of the patients, 72% had Crohn’s disease, and 28% had ulcerative colitis. There were eight (8%) patients who experienced at least one thrombotic event (six patients with deep venous thrombosis (DVT) and two patients with pulmonary embolism (PE)). A family history of DVT was found in 5% of the patients, while a family history of PE was found in 4%. After adjusting for age and gender, the only statistically significant predictor of thrombosis in IBD patients was a family history of thrombotic events (relative risk (RR) = 9.22, 95% confidence interval (CI) = 2.10-40.43) [1].

A systematic review and meta-analysis were done to see whether systemic corticosteroids and anti-tumor necrosis factor-alpha (TNF-α) medications increase the incidence of VTE in IBD patients. They identified 817 records, of which eight observational studies with a total of 58,518 IBD patients were suitable for quantitative synthesis. There were 3,260 thromboembolic incidents in all. Compared to IBD patients who did not take steroid therapy, systemic corticosteroids were linked to a significantly greater probability of VTE complications. In contrast, treatment with anti-TNF-α agents resulted in a fivefold lower incidence of VTE compared to steroid therapy [7].

This study aims to determine the demographic data, prevalence, clinical presentations, diagnosis image, and management of IBD patients with thromboembolic events, define associated risks, and encourage further studies that contribute to prevention measures.

## Materials and methods

Study design, area, population, and sampling

This study is a retrospective, cross-sectional study conducted at King Fahad Specialist Hospital (KFSH) in Buraydah, Al-Qassim, Saudi Arabia. All patients diagnosed with IBD and thromboembolic events between January 1, 2020, and January 1, 2022, were included in the study. The selection of sampling was made by convenience sampling. All subjects who meet the inclusion criteria were included. The inclusion criteria were both genders aged up to 16 years old. The exclusion criteria were those aged below 16 years old and Individuals out of the Al-Qassim region.

Data collection methods

We performed a retrospective chart review of patients diagnosed with inflammatory bowel disease with thromboembolic disease who were seen at KFSH between January 1, 2020, and January 1, 2022. Data were obtained from hospital inpatient medical files. We extracted demographic data on age, gender, area of residency, diagnosis (UC or CD), duration of the disease, clinical features, history of risk factors (old age, extensive diseases, colorectal surgery, immobilization, medications (e.g., corticosteroids and tofacitinib), pregnancy, family or personal history of VTE, and smoking), history of comorbidities (hypertension (HTN), diabetes mellitus (DM) type 1 and 2, chronic kidney disease (CKD), dyslipidemia, cancer, and hematological diseases), methods of diagnosis (Doppler ultrasonogram (US), magnetic resonance imaging (MRI), magnetic resonance angiography (MRA), computed tomography (CT), and vascular angiography), treatments used in the initial presentation of thromboembolic events (warfarin, heparin, clopidogrel, and interventional), and general use of anticoagulant prophylactic in IBD patients. The needed data were entered in an Excel (Microsoft Corp., Redmond, WA, USA) sheet and saved in one secured, password-protected laptop to ensure data safety.

Data management and analysis plan

All values were analyzed using Statistical Package for the Social Sciences (SPSS) Statistics version 23.0 (IBM Corp., Armonk, NY, USA). In addition, continuous data were displayed as mean, mode, and standard deviation, while descriptive statistics were performed with categorical data presented as numbers, percentages, and frequencies. To assess the association of the variables, we used chi-square and t-tests. A p-value < 0.05 was considered statistically significant.

Ethical considerations

Ethical approval was obtained from the Qassim Research Ethics Committee. All the collected data was kept private. Only the researchers can access the information of the patients’ medical records.

## Results

Characteristics of the respondents

A total of 187 participants were included in the current study. The mean age of the participants ± standard deviation was 28.7 ± 10.8 years old. A total of 107 (57.2%) participants were males, and 80 (42.8%) were females. Moreover, 186 (99.5%) were of Saudi Arabian nationality, and one (0.5%) was of non-Saudi nationality. Of the participants, 121 (64.7%) were diagnosed with Crohn’s disease, 56 (29.9%) were diagnosed with ulcerative colitis, and 10 (5.3%) were diagnosed with indeterminate colitis. The duration of the disease was 1-5 years in 156 (83%) participants, 6-10 years in 28 (15%) participants, and more than 10 years in about three (1.6%) participants (Table 1).

**Table 1:** Demographic and clinical characteristics of the respondents (n = 187) SD: standard deviation

Variable	Mean ± SD	Range
Age (years)	28.7 ± 10.8	15-65
	Frequency	Percentage
Gender		
Male	107	57.2%
Female	80	42.8%
Nationality		
Saudi	186	99.5%
Non-Saudi	1	0.5%
Diagnosis		
Ulcerative colitis	56	29.9%
Crohn’s disease	121	64.7%
Indeterminate colitis	10	5.3%
Duration of the disease		
1-5 years	156	83.4%
6-10 years	28	15%
More than 10 years	3	1.6%

Prevalence, risk factor, clinical picture, diagnosis, and management of IBD patients with thromboembolic events

**Figure 1:**
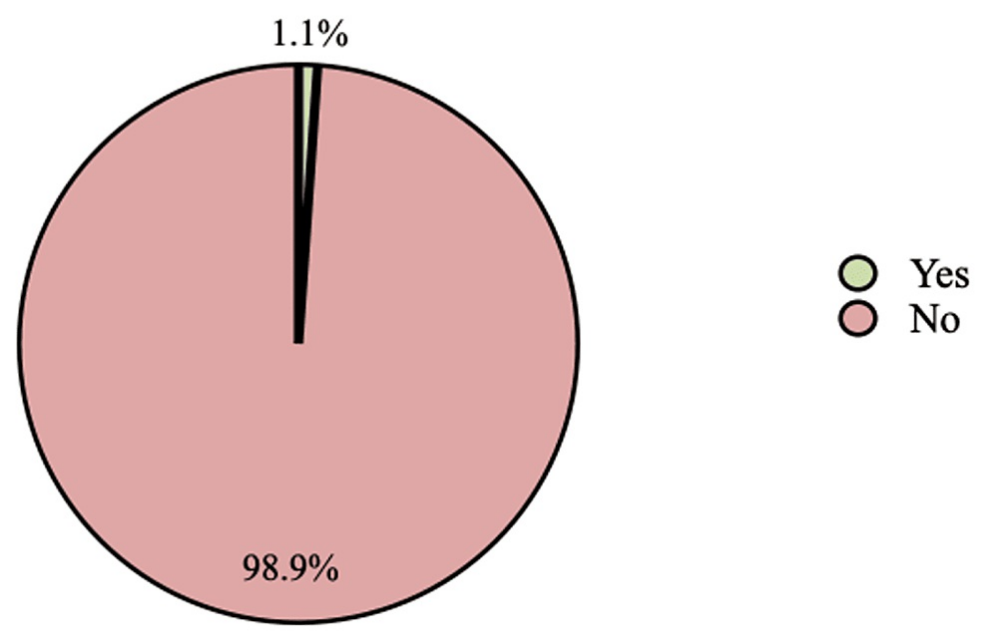
Prevalence of thromboembolic events among inflammatory bowel disease patients

Among IBD patients, thromboembolic events were found in two (1.1%), including interval resolution middle and left portal vein and inferior mesenteric vein thrombosis (0.5%) and DVT (0.5%), whereas about 185 (98.9%) participants were with no previous history of thromboembolic events (Figure 1).

Regarding the history of risk factors and their association with thromboembolic events, old age was not significantly associated with thromboembolic events (p = 1.000). Active or extensive disease was also not significantly associated with thromboembolic events (p = 0.400). No significant association was found between colorectal surgery and thromboembolic events (p = 0.164). Immobilizations were not significantly associated with thromboembolic events (p = 1.000). There was no difference between medications (corticosteroids and tofacitinib) in terms of thromboembolic events (p = 1.000). However, medications were the most common risk factors among 94 patients with a percentage of 50.3%, with the absence of thromboembolic events in 93 patients and presence in only one patient. This indicates that medications do not play direct relation to TEE unless there are other risk factors. No significant association was found between pregnancy and thromboembolic events (p = 0.053). There was a significant association between family or personal history of TED and thromboembolic events (p = 0.021). Smoking was not associated with thromboembolic events (p = 1.000). No significant association was found between overweight and thromboembolic events (p = 1.000) (Table 2).

**Table 2:** History of risk factors and their association with thromboembolic events TED: thromboembolic disease

Risk factors	Total	Thromboembolic events		p-value
		Present	Absent	
Old age	8 (4.3%)	0 (0%)	8 (4.3%)	1.000
Active or extensive disease	42 (22.5%)	1 (50%)	41 (22.2%)	0.400
Colorectal surgery	16 (8.6%)	1 (50%)	15 (8.1%)	0.164
Immobilization	1 (0.5%)	0 (0%)	1 (0.5%)	1.000
Medications (corticosteroids and tofacitinib)	94 (50.3%)	1 (50%)	93 (50.3%)	1.000
Pregnancy	5 (2.7%)	1 (50%)	4 (2.2%)	0.053
Family or personal history of TED	2 (1.1%)	1 (50%)	1 (0.5%)	0.021
Smoking	10 (5.3%)	0 (0%)	10 (5.4%)	1.000
Overweight	4 (2.1%)	0 (0%)	4 (2.2%)	1.000
No risk factors	63 (33.7%)	0 (0%)	63 (34.1%)	0.551

**Figure 2:**
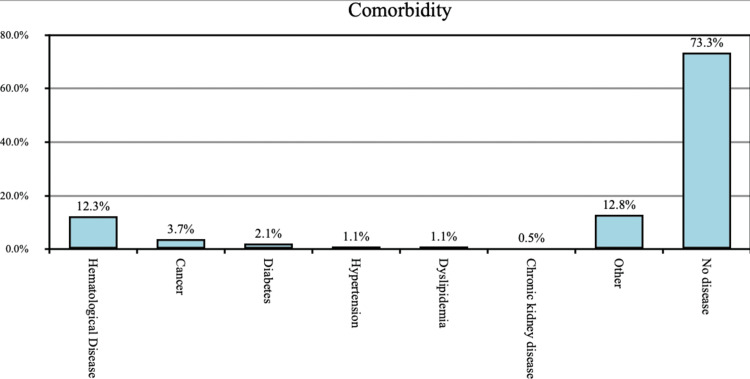
History of comorbidity

In regard to the history of comorbidities among the participants, the majority of the participants (73.3%) were with no history of comorbid conditions, but of the participants, about 12.3% had hematological diseases, 3.7% had cancer, 2.1% had diabetes, 1.1% had hypertension, 1.1% had dyslipidemia, 0.5% had chronic kidney disease, and 12.8% had other diseases such as asthma, thyroid diseases, gonarthrosis, acute appendicitis, cardiac arrhythmia, calculus of the ureter, and gastritis (Figure 2).

**Figure 3:**
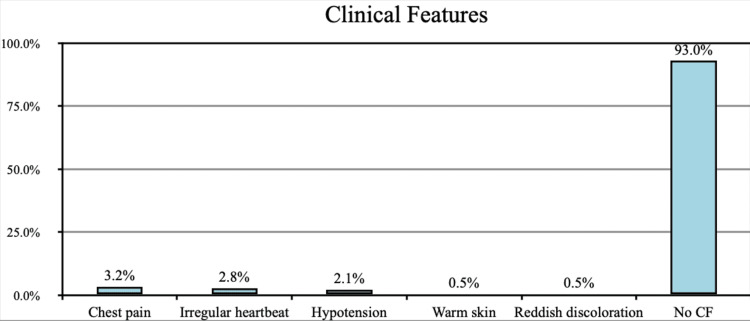
Clinical features CF: clinical features

The most reported clinical symptoms were chest pain as reported by 3.2% of the participants, followed by irregular heartbeat (2.8%), hypertension (2.1%), warm skin (0.5%), and reddish discoloration (0.5%). Of the participants, 93% were with no clinical features (Figure 3).

**Figure 4:**
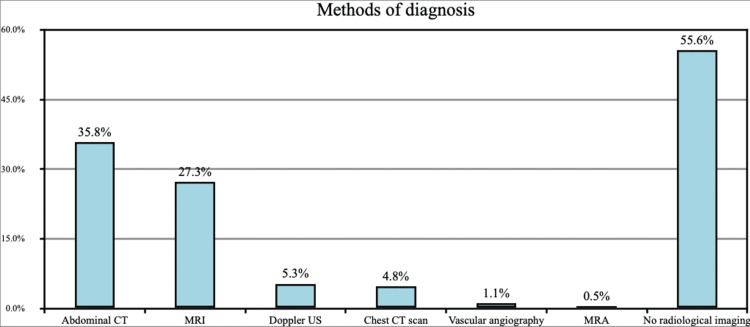
Methods of diagnosis CT: computed tomography; MRI: magnetic resonance imaging; US: ultrasound; MAR: magnetic resonance angiography

Abdominal CT was the most reported method of diagnosis as reported by 35.8% of the participants. MRI was the diagnosis method used in about 27.3% of the participants, Doppler ultrasound in 4.8%, chest CT scan in 4.8%, vascular angiography in 1.1%, and MRA in 0.5%. In about 55.6% of the participants, no radiological imaging was done (Figure 4).

**Figure 5:**
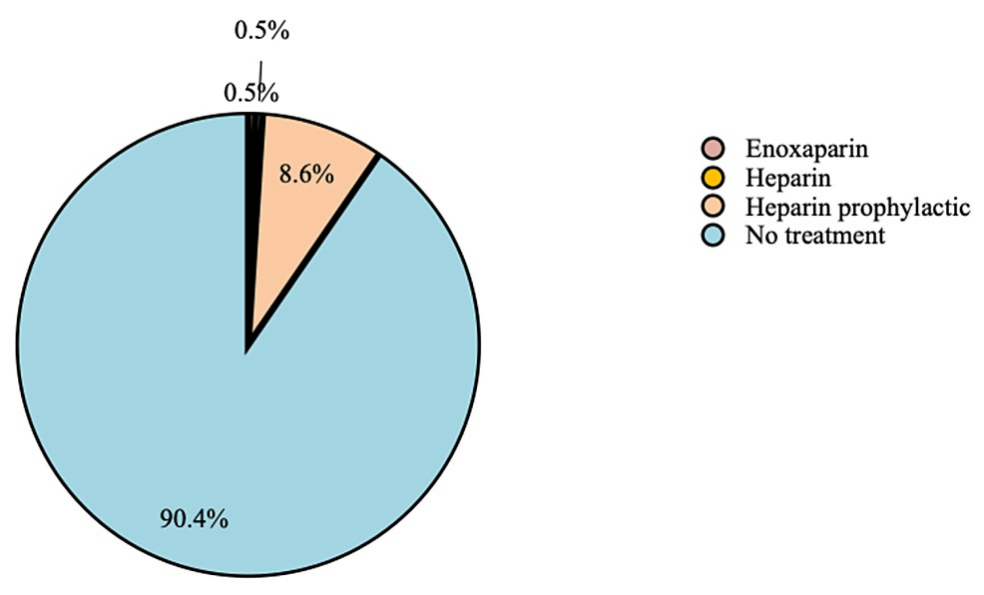
Treatment

About 90.4% did not receive any treatment, about 8.6% used heparin prophylactically, 0.5% used heparin as a treatment, and 0.5% used enoxaparin as a treatment (Figure 5).

**Figure 6:**
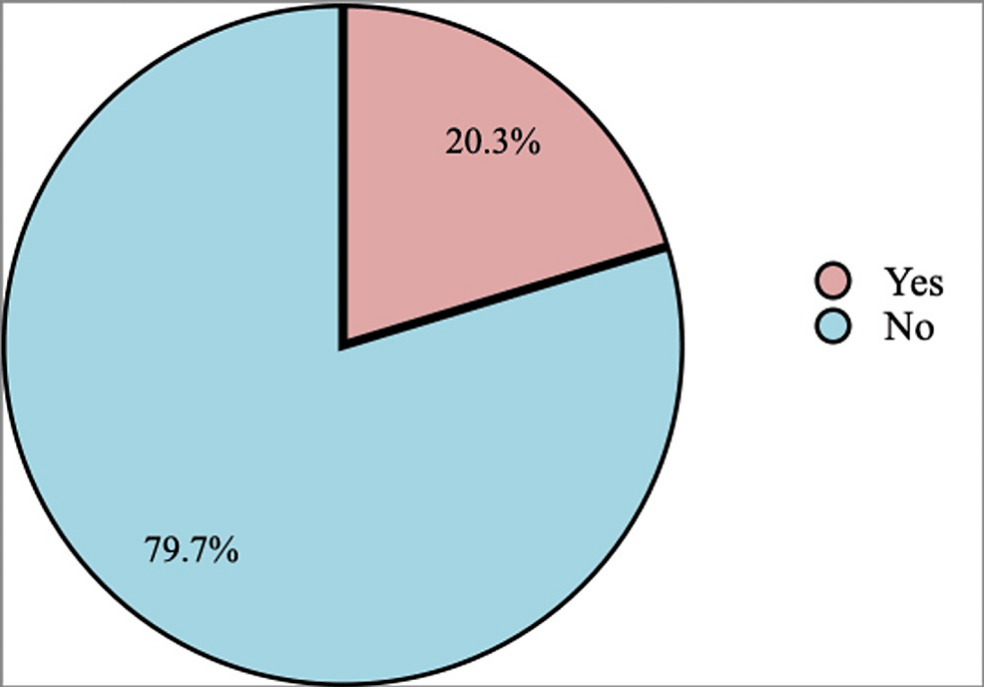
Does the patient take an anticoagulant prophylactic?

About 20.3% of the participants used anticoagulant prophylactic treatment, whereas about 79.7% did not use prophylactic treatment (Figure 6).

Age, gender, and nationality were not found to be significantly associated with thromboembolic events (p = 0.915, 1.000, and 1.000, respectively). Also, diagnosis, duration of the disease, and taking prophylaxis were not found to be significantly associated with thromboembolic events (p = 0.610, 0.305, and 0.366, respectively) (Table 3).

**Table 3:** Association between demographic data, diagnosis, duration, prophylactic, and thromboembolic event development in inflammatory bowel disease patients SD: standard deviation

Variable	Thromboembolic events		p-value
	Present	Absent	
	Mean ± SD		
Age (years)	29.5 ± 0.7	28.7 ± 10.8	0.915
	Number (%)		
Gender			
Male	1 (0.9%)	106 (99.1%)	1.000
Female	1 (1.3%)	79 (98.8%)	
Nationality			
Saudi	2 (1.1%)	184 (98.9%)	1.000
Non-Saudi	0 (0%)	1 (100%)	
Diagnosis			
Ulcerative colitis	0 (0%)	56 (100%)	0.610
Crohn’s disease	2 (1.7%)	119 (98.3%)	
Both ulcerative colitis and Crohn’s disease	0 (0%)	10 (100%)	
Duration of the disease			
1-5 years	1 (0.6%)	155 (99.4%)	0.305
6-10 years	1 (3.6%)	27 (96.4%)	
More than 10 years	0 (0%)	3 (100%)	
Taking a prophylactic			
Yes	1 (2.6%)	37 (97.4%)	0.366
No	1 (0.7%)	148 (99.3%)	

## Discussion

Patients with inflammatory bowel disease may be at increased risk of developing thromboembolic events, which have variable morbidity and mortality. Therefore, assessing the prevalence of thromboembolic events in IBD patients could result in a more accurate determination of disease status and to which extent thromboembolic events are common, which in turn could result in a more accurate determination of the responsible factors in the subsequent studies [8].

Thromboembolic disease is caused by several genetic and acquired factors. The existence of more than one genetic factor may increase the risk of developing recurrent thrombotic events. Several factors can influence increased TED risk, including disease activity, hospitalization, age, pregnancy, medications, surgery, and genetics [9].

This study found that two (1.1%) out of 187 patients had a thrombotic event. One of these two patients was pregnant, and she had a personal history of DVT. Prior studies on the genetic hazards of TED in IBD patients have primarily concentrated on monogenic variations such as factor V Leiden deficiency. However, a recent study found that the existence or lack of discrete large effect mutations is not the only factor affecting the general population’s chance of developing TED but also a polygenic risk, which refers to the effect of numerous loci, often of a small individual effect, across the genome [9]. In a study including two extremely sizable population cohorts, the combination of a polygenic risk score (PRS), which pools many genetic TED risk variants, and two monogenic variants was used to assess the risk of TED. Factor V Leiden deficiency and prothrombin G20210A mutation delineated 10% of the individuals with an approximately 2.5-fold increased likelihood of developing TED compared with non-high-risk controls, which is attributed to risk factors, including disease flare, extended disease location, and steroid use; a higher genetic risk was associated with TED events, suggesting that these IBD patients might warrant more aggressive prophylaxis against TED [9].

The current study aimed to identify the prevalence, risk factors, clinical picture, and management of TEE among IBD patients at KFSH in Buraydah, Al-Qassim, Saudi Arabia. The mean age of the participants in this study was found to be 28.7 years old. More than half (57.2%) of the participants were males, and 42.8% were females. Nearly all (99.5%) participants were of Saudi Arabian nationality.

About two-thirds (64.7%) of the participants were diagnosed with CD, 29.9% of the participants were diagnosed with ulcerative colitis, and 5.3% of the patients were diagnosed with both CD and UC; this was found to be contradictory to that reported in the study conducted by Lynch et al., who found that the prevalence of UC is greater than CD [10].

The prevalence of thromboembolic events among IBD patients was found to be 1.1%, mostly in the interval resolution middle and left portal vein and inferior mesenteric vein. The thromboembolic events noted were thrombosis (0.5%) and DVT (0.5%), and similar findings were reported in the parallel study conducted by Arvanitakis et al., in which the prevalence was found to be between 1.3% and 7.7% [11]. Also, one congruent study reported a similar prevalence [8].

Concerning the history of risk factors and their association with thromboembolic events, old age was not significantly associated with thromboembolic events in IBD patients. Active or extensive disease was also not found to be significantly associated with thromboembolic events in IBD. No significant association was found between colorectal surgery and thromboembolic events in IBD. Immobilization was not found to be significantly associated with thromboembolic events. There was no difference between medications (corticosteroids and tofacitinib) in terms of thromboembolic events. No significant association was found between pregnancy and thromboembolic events. No significant association was also found between overweight and thromboembolic events. The abovementioned associations were found to be in contradiction to a study conducted by Cheng and Faye, who demonstrated that the known risk factors of thromboembolic events such as old age, pregnancy, extensive disease, and colorectal cancer were found to be significantly associated with thromboembolic events in IBD patients [8].

Regarding the history of comorbidities among the participants, more than two-thirds (73.3%) of the participants were with no history of comorbid conditions. About 12.3% of the participants had hematological diseases, 3.7% had cancer, 2.1% had diabetes, 1.1% had hypertension, 1.1% had dyslipidemia, 0.5% had chronic kidney disease, and 12.8% had other diseases; similar findings were found in the congruent study carried out by Bähler et al., in which these findings were also mentioned [12].

The most commonly reported clinical symptoms were chest pain as reported by 3.2% of the participants. Of the participants, 2.8% mentioned irregular heartbeat, 2.1% reported hypertension, 0.5% reported warm skin, and 0.5% reported reddish discoloration, and this was found to be consistent with the study conducted by Gong et al., in which chest pain and chest tightness were also the most commonly reported clinical features of thromboembolic event [13].

Abdominal CT was the most frequently reported method of diagnosis of thromboembolic events as reported by 35.8% of the participants. MRI was the diagnosis method in 27.3% of the participants, Doppler ultrasound in 4.8%, chest CT scan in 4.8%, vascular angiography in 1.1%, and MRA in 0.5%; a similar finding was reported in the parallel study carried out by Wang et al., in which imaging and D-dimer were the most reported methods of diagnosis [14].

Considering the treatment of thromboembolic events, about 8.6% of the participants used heparin prophylactically, 0.5% used heparin as a treatment, and 0.5% used enoxaparin as a treatment. Moreover, 90.4% did not receive any treatment. About 20.3% of the participants used prophylactic treatment generally, which was consistent with the reported finding in the congruent study conducted by Kaddourah et al., in which 39.7% of the patients with IBD received venous thromboembolism prophylaxis [15].

The association between sociodemographic characteristics of the participants and age, gender, and nationality were not significantly associated with thromboembolic events. Also, diagnosis, disease duration, and prophylaxis were not found to be significantly associated with thromboembolic events; similar findings were reported in the study conducted by Bulbul et al., in which age and gender were not found to be associated with thromboembolic events [16].

Limitations

This study is based on previously recorded data, so it is prone to registry bias, and some critical data may be missing. Besides, controlling confounders is difficult. Furthermore, the study was restricted to residents of Al-Qassim, Saudi Arabia. As a result, it may not accurately reflect the results of the entire Saudi population. Moreover, a low incidence rate is a major limitation in assessing risk factors. We encountered some difficulties in assessing genetic factors in TED related to IBD patients.

## Conclusions

Despite IBD being one of the emerging health concerns in the Kingdom of Saudi Arabia, the records showed that the prevalence of thromboembolic events was found to be lower when compared to the prevalence reported in the relevant studies. There was no difference in factors affecting the development of thromboembolic events between IBD patients and the general population.
